# Ectopic Unilateral Ear Pit in an Otherwise Well-Appearing Child: A Case Report and Literature Review

**DOI:** 10.7759/cureus.39720

**Published:** 2023-05-30

**Authors:** Henry H Nguyen, Sydney Martin, Leslie Jabine

**Affiliations:** 1 Pediatrics, University of Illinois at Chicago, Chicago, USA; 2 Pediatrics, The University of Chicago Medicine, Chicago, USA

**Keywords:** external ear defects, external ear deformities, external ear abnormalities, preauricular sinus, preauricular pit, ear pit

## Abstract

Ear pits are a common congenital abnormality that is incidentally found on routine examinations. However, it is not well documented how many are found outside of their classical location or if these ectopic locations put patients at increased risk of having a hearing impairment, renal anomalies, genetic syndromes, or infection. Clinicians should be aware of the current guidelines for recognizing, screening, and evaluating for these risks in patients with ear pits, regardless of location.

## Introduction

Preauricular pits, also known as preauricular fistulas, preauricular tracts, preauricular cysts, ear pits, or ear sinuses, were first described in 1864 by Heusinger on a routine physical exam [1]. These common congenital abnormalities are small invaginations of stratified squamous epithelium, typically found on or adjacent to the auricle of the external ear. They are usually asymptomatic but are prone to infection. The estimated incidence of preauricular pits is 0.1-0.9% in the United States, occurring predominantly in Asian-American children (10%), African-American children (5%), and Caucasian-American children (1%) [2-6]. Ear pits can be found unilaterally or bilaterally. They can occur in isolation or in association with certain genetic syndromes, such as Branchio-Oto-Renal (BOR) syndrome. Patients with ear pits are also at increased risk of having hearing loss and/or renal abnormalities. Although current literature suggests no workup is needed for isolated ear pits, auditory testing and renal ultrasound may be useful for patients with an increased risk of hearing loss and renal dysfunction [4,6]. Medical and surgical management is typically reserved for patients with infected pits. In this article, we will briefly summarize the epidemiology, embryology, clinical significance, evaluation, and management of ear pits and address the question of whether variant ear pits have an increased relationship with hearing loss, kidney anomalies, and genetic syndromes.

## Case presentation

A two-year-old male with well-controlled, moderately persistent asthma and eczema was brought into our pediatric clinic by his mother after she found a hole in his left outer ear while giving him a bath. This was the first time the mom noticed it, and there were no documented ear pits on prior well-child visits upon chart review. Mom reported no issues or changes to his hearing; no otalgia, fever, or drainage from the orifice. There was no recent trauma to the ear. The patient had normal speech development, and there was no history of frequent otitis media or externa. There were also no urinary symptoms or histories of urinary infections. There was no family history of hearing loss, ear pits, or genetic syndromes.

**Figure 1:**
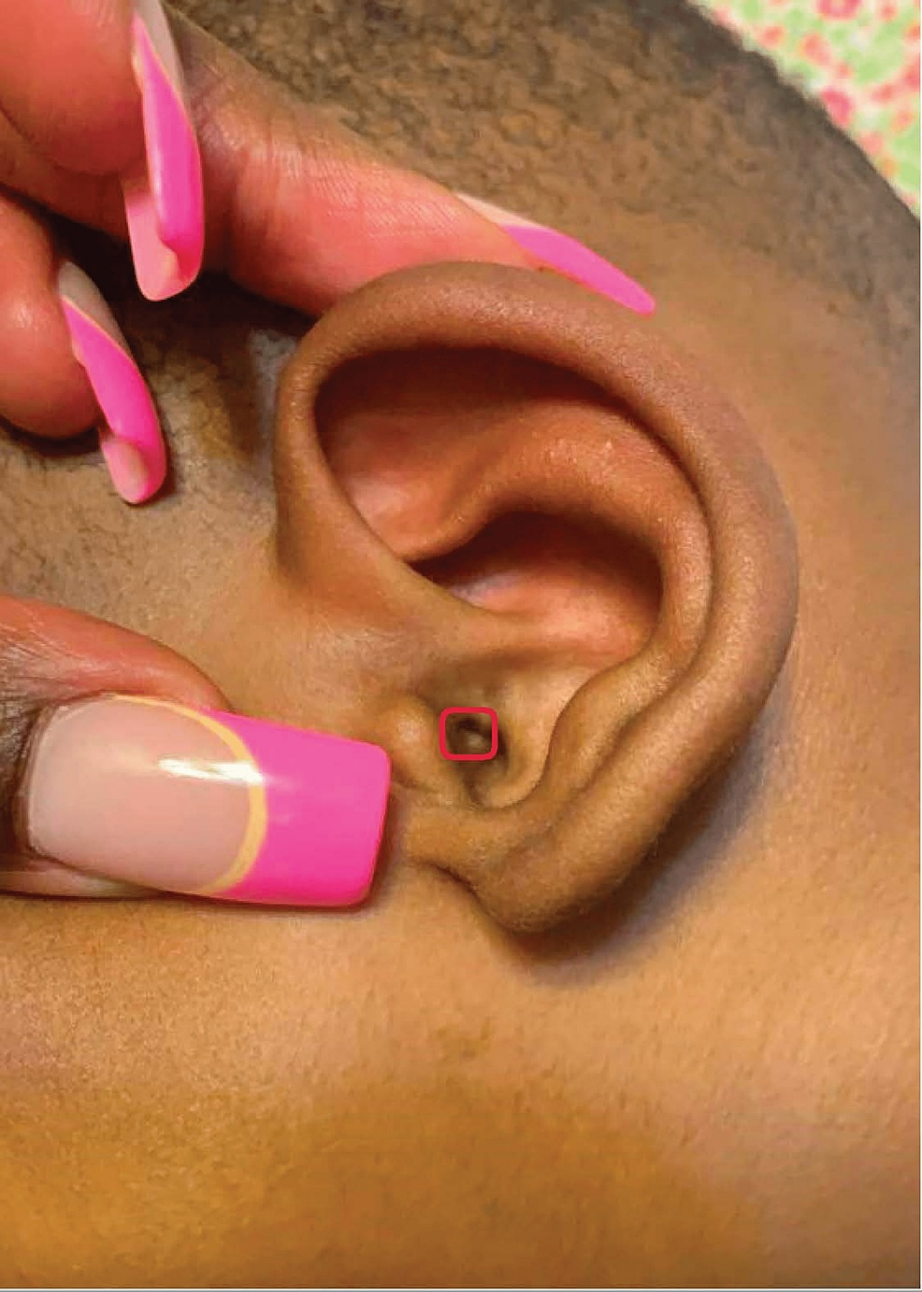
Case patient with ectopic ear pit Verbal consent was obtained

On physical exam, the patient was afebrile and well-appearing. He had no apparent dysmorphic features. There was a small hole similar to an ear pit in the concha, just inside the external auditory meatus on the anterior portion of the external auditory canal of the left ear, without any drainage or surrounding inflammation (Figure 1). There were no other preauricular sinuses in either ear. The ear canals and tympanic membranes of both ears were normal.

The mother was advised that the appearance of the hole was similar to a preauricular sinus but in a variant location, likely benign, and not associated with any syndromes since his hearing was intact. The mother was also advised that sinus tracts can become infected, so anticipatory guidance was given to monitor for signs of infection, and the patient was referred to a pediatric ENT for further evaluation.

ENT’s comprehensive audiology evaluation was unremarkable, showing normal tympanometry, acoustic reflexes, and otoacoustic emissions bilaterally. In addition, a microscopic exam of the ear and the tympanic membrane was normal in the ENT clinic. ENT further reiterated anticipatory guidance for signs of recurrent infection, and an audiology evaluation was recommended on an as-needed basis.

## Discussion

Epidemiology

Ear pits are a common congenital malformation and can occur either sporadically or hereditarily [2,4,7]. The incidence varies globally and is estimated to be 0.1-0.9% in the United States, occurring in 10% of Asian-American, 5% of African-American, and 1% of Caucasian-American children [2-6]. Both sexes are equally affected [8]. They are unilateral in over 50% of cases, more often on the right ear, and bilateral in 25-50% of cases [2-7].

Presentation

**Figure 2:**
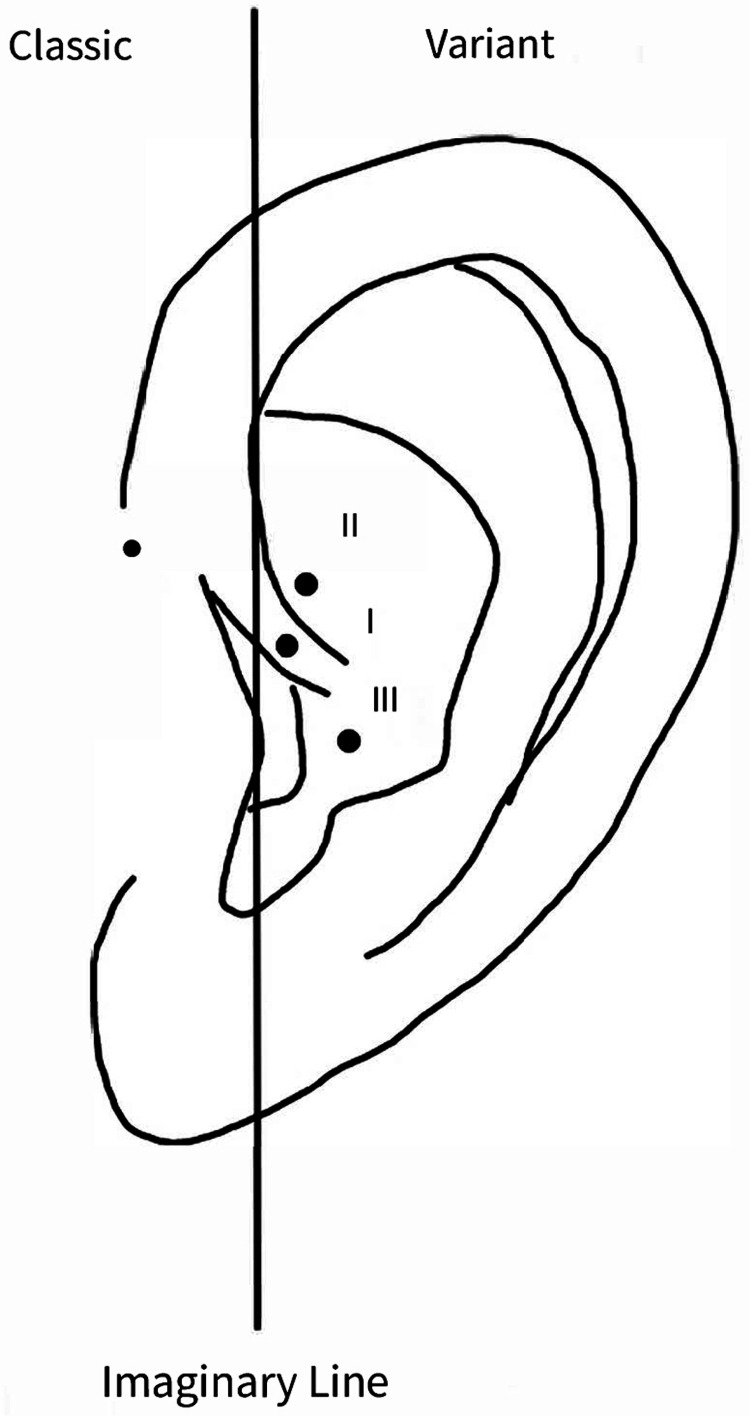
Classification of ear pit location Classic: anterior to the helix and superior to the tragus. Variant: type I - middle of the crus; type II - superior to the crus; type III - at the cymba concha. Image Credits: Henry Nguyen, DO MS, author.

Ear pits are small indentations or openings less than 3 mm from the external ear [6]. They are commonly found incidentally in newborns, but because of their small size and lack of symptoms, they are often missed on routine examinations and cause parental distress because of their cosmetic appearance. Pits have been categorized based on their location in relation to an imaginary tragal vertically extended line into classical types and variant types (Figure 2) [3,7,9]. The classical type is usually located anterior to the helix and superior to the tragus (preauricular) [6]. The variant type is uncommon and usually located post-auricularly, either in the middle of the crus (type 1), superior to the crus (type 2), or at the cymba concha (type 3) (Figure 2) [3,7]. Ear pits that do not conform to the classical or variant types are extremely rare, found in less than ten reported cases [6,7,9,10].

Embryology and histology

**Figure 3:**
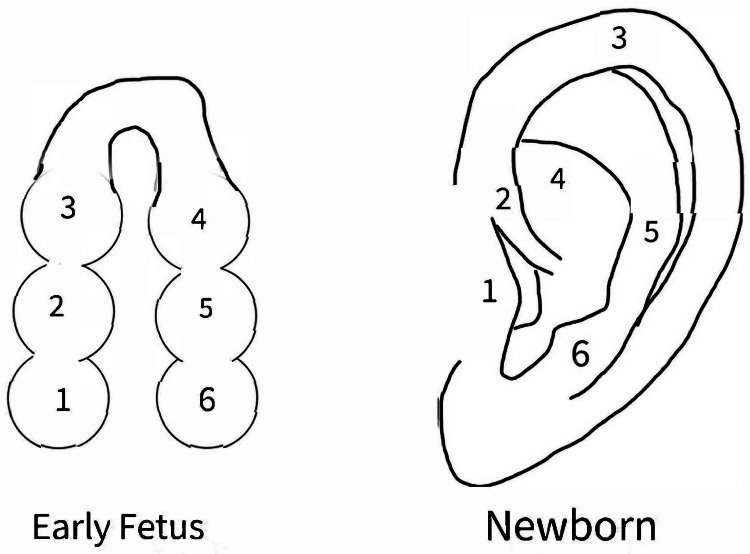
Hillocks of His and its development into the human ear (1) Tragus, (2) Helix Crus, (3) Helix, (4) Antihelix, (5) Antitragus, (6) Lobule and Inferior Helix. Image Credits: Henry Nguyen, DO MS, author.

The embryogenesis of ear pits is not yet known. From the sixth week of gestation, tissue from the first and second branchial clefts develops into six auditory hillocks (known as the hillocks of His; three from the caudal edge of the first branchial cleft and three from the cephalic edge of the second branchial cleft), which unite to form the external ear within the next few weeks of embryogenesis (Figure 3) [2-9,11-15]. Currently, there are three theories for the development of ear pits: incomplete fusion of the first three auditory hillocks from the first branchial cleft, isolation of ectodermal folds during auricle formation, and defective closure of the most dorsal part of the first branchial cleft [2-9,12,14,15].

The inner surface of ear pits consists of stratified squamous epithelium [3,13]. Ear pits can be filled with smegma-like material and can contain sebaceous glands, sweat glands, and hair follicles [6].

Clinical significance

Ear pits are usually isolated and asymptomatic. However, pits can be superficial or have tracts that can vary in length, branch, and follow a tortuous course [4-10]. This can result in the formation of a cyst that can get infected or an abscess. The most common infectious pathogens are Staphylococcal species, followed by Proteus, Streptococcus, and Peptococcus species [4-6,9]. In addition, these tracts can extend to the facial nerve and parotid gland, potentially causing facial nerve damage/paralysis and parotitis [6].

Ear pits can appear concurrently with some congenital anomalies and syndromes associated with hearing impairment, occurring in as many as 15-30% of cases [2]. In one cross-sectional study of 68,484 infants, hearing impairment was found to be seven times more common among infants with isolated ear pits compared to infants without ear pits [12]. Even though hearing impairment is due to an inner ear malformation, which originates from different embryonic tissue than the external ear, multiple ear malformations may be possible due to the proximity of the different tissues and because the inner and external ear develop at the same time of embryogenesis [12].

About 2.6% of patients with ear pits are also associated with some syndromes with renal anomalies [2,11]. Although the incidence of kidney anomalies in patients with isolated ear pits is similar to that of the general population, they may be the first indication of BOR syndrome. It is characterized by hearing loss (70-93% of affected patients; one of the most common causes; approximately 2% of all childhood hearing loss), ear pits, branchial cysts or tracts, malformed ears, and kidney anomalies [16,17]. Ear pits and kidney anomalies can occur in several other syndromes, including CHARGE syndrome, Townes-Brocks syndrome, Nager syndrome, Miller syndrome, the oculo-auriculo-vertebral spectrum, and diabetic embryopathy, to name a few [18].

Evaluation

Patients with ear pits should have a formal audiologic evaluation. The risk of permanent hearing loss in these patients is five times that of the general population [2,6,12].

Routine renal ultrasound for patients with isolated ear pits is up for debate. A study involving 17,286 healthy newborns with isolated preauricular tags and pits did not find a significant association with urinary tract abnormalities, with those found being mostly common congenital anomalies such as mild pyelectasis or hydronephrosis [18]. However, due to the high incidence of renal anomalies in syndromes with auricular malformations, renal ultrasound should be obtained in children with ear pits accompanied by any of the following [2,6,18]: (1) other malformations or dysmorphic features; (2) family history of deafness or auricular or kidney malformations; (3) maternal history of gestational diabetes.

Treatment

Ear pits generally do not require intervention. However, patients require appropriate antibiotics when infected, incision and drainage if an abscess is present, and surgery for recurrent or persistent infections [4,5,8-10,14]. In one study, about 75% of subjects up to a median age of 19 years remained asymptomatic, and 28% of symptomatic patients experienced symptoms after age 16 [2]. Surgery includes methylene blue staining and probing to assess the extent of the tract course, followed by excision of the pit, its tract/cyst, and the cartilage at the root of the helix en bloc to avoid recurrence [4-6,14,15]. Recurrence typically occurs due to incomplete resection of sinus tracts due to potential branching between 19% and 40% [4,5,14,15]. Details of the different surgical methods are excluded from the discussion of this case report.

## Conclusions

Ear pits are incidentally found as congenital abnormalities that are typically asymptomatic and require no intervention. Most pits are classically located anterior to the helix and superior to the tragus, but in rare cases, they are located elsewhere on or around the ear. Our patient’s ear pit would be categorized as a type 3 variant pit. Pits are associated with hearing loss and renal anomalies, as seen in various genetic syndromes, but there have been no studies that determine the prevalence of variant types or if they have a greater association compared to classical locations. These questions warrant further study. Regardless of location, patients with ear pits should have a formal audiologic evaluation. Given the high incidence of renal anomalies in syndromes with auricular malformations, renal ultrasound should also be considered for patients with ear pits and urinary symptoms or increased risk of kidney dysfunction, along with (1) other malformations or dysmorphic features, (2) family history of deafness, auricular, or kidney malformations, or (3) maternal history of gestational diabetes. Infected ear pits should be treated with antibiotics. Abscesses should be drained. Surgical exploration and complete excision of the sinus tract are the treatments of choice for recurrent and complex infections.
